# Extranodal lymphoma of the head and neck: a pictorial essay

**DOI:** 10.1590/0100-3984.2017.0232

**Published:** 2019

**Authors:** Pinar Gulmez Cakmak, Gülsüm Akgün Çağlayan, Furkan Ufuk

**Affiliations:** 1 Department of Radiology, Pamukkale University Medical Center, Denizli, Turkey.

**Keywords:** Intraocular lymphoma, Tomography, X-ray computed, Magnetic resonance imaging, Head and neck neoplasms, Lymphoma, Linfoma intraocular, Tomografia computadorizada, Ressonância magnética, Neoplasias de cabeça e pescoço, Linfoma

## Abstract

Primary extranodal lymphoma is defined as a lymphoma at a solitary extranodal site, with or without involvement of the lymph nodes. The clinical and radiological features of extranodal lymphoma have been documented in recent studies. In this pictorial essay, we reviewed imaging findings of extranodal lymphoma in the head and neck region.

## Abstract (pt)

Linfoma extranodal primário é definido como um sítio extranodal solitário de linfoma, com ou sem envolvimento dos gânglios linfáticos. As características clínicas e radiológicas do linfoma extranodal foram documentadas em estudos recentes. Neste ensaio revisamos os achados de imagem do linfoma extranodal da região da cabeça e pescoço.

## INTRODUCTION

Lymphoma is the most common nonepithelial malignancy of the head and neck region. Non-Hodgkin lymphomas are found in extranodal regions outside the lymphoid system in 40% of cases. The clinical features, computed tomography (CT) findings, and magnetic resonance imaging (MRI) findings of extranodal lymphomas in the head and neck region have been described^(^^1^^)^. Extranodal lymphoma of the head and neck can affect the paranasal sinuses, nasal cavity, salivary glands, thyroid gland, or orbit.

## ORBITAL LYMPHOMA

Lymphoid tumors usually occur in lymph nodes but can involve extranodal sites, including the orbits. Ocular lymphoma is a malignant lymphoproliferative neoplasm that affects the eyelid, conjunctiva, orbit, or lacrimal gland, accounting for 30% of orbital tumors^(^^2^^**,**^^3^^)^. The most common types of ocular lymphoma are extranodal marginal zone lymphoma and mucosa-associated lymphoid tissue lymphoma, other types including follicular cell, mantle cell, diffuse large B-cell, and lymphoplasmacytic lymphoma^(^^4^^)^. Lymphoproliferative disease of the orbit encompasses a spectrum from benign (reactive) lymphoid hyperplasia to malignant lymphoma, the most common clinical symptoms being exophthalmos, eyelid swelling, and diplopia^(^^4^^)^.

**Figure 1:**
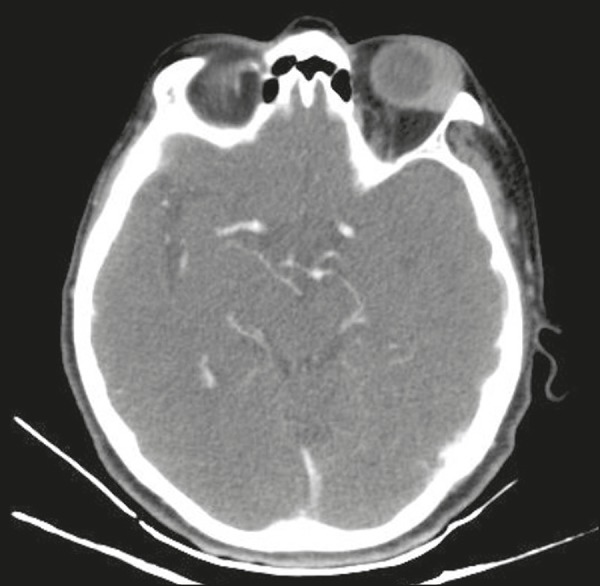
Contrast-enhanced axial CT scan showing lymphoma of the left ocular adnexa. The mass is located in the upper-outer quadrant of the left orbit and shows contrast enhancement.

**Figure 2:**
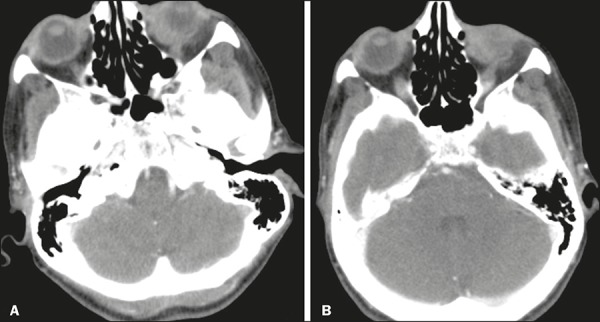
A,B: Contrast-enhanced axial CT scan showing lymphoma of the left ocular adnexa. The mass is located in the upper-outer quadrante of the left orbit and shows contrast enhancement.

**Figure 3:**
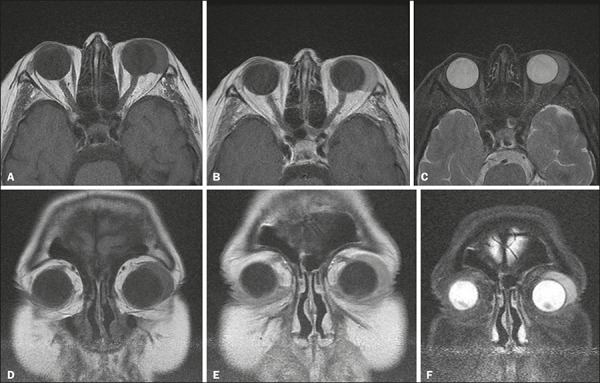
Axial and coronal MRI scans showing ocular lymphoma—axial T1W image (**A**), contrast-enhanced axial T1W image (**B**), axial T2W image (**C**), coronal T1W image (**D**), contrast-enhanced coronal T1W image (**E**), coronal T2W image (**F**)—in the upper-outer quadrant of the left orbit.

Orbital lymphoma is seen as a well-defined soft-tissue mass in the orbit^(^^1^^)^. It is typically located near the lacrimal gland in the upper outer quadrant of the orbit. A soft-tissue mass surrounding the extraocular muscles is helpful in distinguishing lymphomas from other orbital masses^(^^2^^)^. On CT, orbital lymphoma is isodense to the extraocular muscles (Figures 1 and 2). On contrast-enhanced CT scans, there is limited contrast enhancement. On MRI, in comparison with the extraocular muscles, orbital lymphomas show signal intensities that are isointense or hypointense on T1-weighted (T1W) and isointense or hyperintense T2-weighted (T2W) images (Figure 3), as well as showing homogeneous contrast enhancement on contrast-enhanced MRI scans and restricted diffusion on diffusion-weighted images^(^^2^^**,**^^5^^)^.

## THYROID LYMPHOMA

Primary thyroid lymphoma is rare, accounting for only approximately 5% of all thyroid malignancies and less than 3% of all extranodal lymphomas^(^^6^^)^. It is a rare malignancy that is intrinsically associated with Hashimoto’s thyroiditis. It presents as a goiter, accompanied by pressure symptoms, and it is necessary to make a rapid, accurate diagnosis. The most common clinical presentation of thyroid lymphoma is a rapidly growing goiter and compressive symptoms, including dyspnea, stridor, hoarseness, and dysphagia^(^^7^^)^. The histological subtypes of thyroid lymphoma are diffuse large B-cell lymphoma and mucosa-associated lymphoid tissue lymphoma.

Ultrasound is the imaging modality of choice for thyroid lymphoma and can typically show one of three patterns^(^^6^^**,**^^8^^)^: nodular, diffuse, or mixed. CT can be used in order to assess involvement of surrounding structures and to facilitate accurate surgical planning, as well as to diagnose cervical and mediastinal lymph node disease. In one study of patients with primary thyroid lymphoma, Kim et al.^(^^9^^)^ reported that none of the patients showed tumor invasion into the adjacent common carotid artery or surrounding muscle, as was confirmed during surgery.

**Figure 4:**
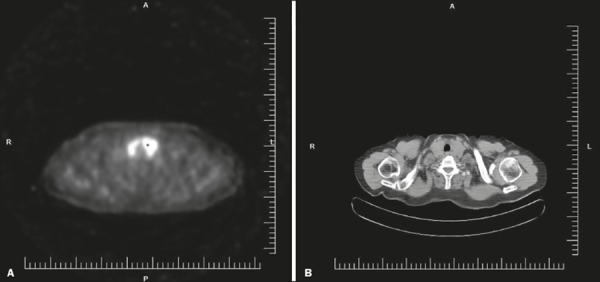
^18^F-FDG-PET/CT scan of a patient with primary thyroid lymphoma in the left lobe of the thyroid gland.

Thyroid lymphoma shows a mainly homogeneous, expansile growth pattern. Although it can present as multiple nodules, it presents as a solitary mass in 80% of cases^(^^10^^)^. Necrotic degeneration and calcification are rare in a thyroid lymphoma. On MRI, patients usually present with a rapidly enlarging thyroid mass and obstructive symptoms related to compression of the aerodigestive tract, and the signal intensities on T1W and T2W images are isointense relative to the neck muscles. For staging, restaging or assessing the response to treatment in thyroid lymphoma, ^18^F-fluorodeoxyglucose positron emission tomography/CT (^18^F-FDG-PET/CT) can be useful^(^^11^^)^ (Figure 4).

## SINONASAL LYMPHOMA

**Figure 5:**
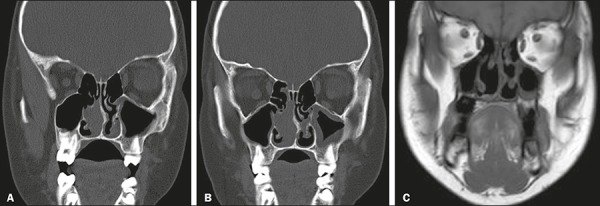
**A:** Coronal CT scan of sinonasal lymphoma showing a soft-tissue mass that is isodense to the muscle, in the left nasal cavity. **B:** Coronal CT scan, obtained one month later, showing a slight increase in the size of the mass. **C:** Coronal T1W MRI after surgery.

Hematolymphoid lesions account for approximately 12-17% of all malignant neoplasms in the nasal cavity and paranasal sinuses. Squamous cell neoplasms are the most common sinonasal malignancies^(^^12^^)^. Diffuse large B-cell lymphoma, the most common type of lymphoma, and natural killer/T-cell lymphoma, nasal type, are highly prevalent in Asians^(^^12^^**-**^^14^^)^. In sinonasal lymphoma, the spectrum of clinical symptoms encompasses nasal obstruction, rhinorrhea, epistaxis, postnasal drip, facial swelling, neck mass, fever, and weight loss. The differential diagnoses include sinonasal carcinoma, olfactory neuroblastoma, and Wegener’s granulomatosis. Sinonasal lymphoma presents as a diffuse infiltrative soft-tissue mass extending along the paranasal sinus and nasal cavity. On CT, it appears as a soft-tissue mass that is isodense to muscle tissue (Figure 5). CT is useful for identifying bone destruction and for staging. Due to its high soft-tissue resolution, MRI is used in order to detect the spread of the disease. On T1W images, sinonasal lymphoma shows a signal intensity that is isointense to the soft tissue (Figure 5), whereas it shows a hyperintense signal on T2W images, as well as showing enhancement after contrast administration^15^.

## CONCLUSION

Primary extranodal lymphomas are rare and can mimic other neoplasms or inflammatory conditions. Lymphomas should be included as a major differential diagnosis of a soft-tissue mass and can be identified through the use of CT or MRI.
